# Predictors of contraceptive discontinuation in Rwanda: evidence from demographic and health survey 2019–2020

**DOI:** 10.1186/s40834-024-00282-y

**Published:** 2024-04-25

**Authors:** Harerimana Jean de Dieu, Mugabo Lambert

**Affiliations:** 1 D-Hause Co, Kigali, Rwanda; 2 Kigali, Rwanda

**Keywords:** Contraceptive discontinuation, Family planning, Life table methods, Discontinuation rates, Health concerns, Side effects

## Abstract

**Background:**

Despite advancements, Rwanda continues to face challenges regarding contraceptive discontinuation. The 2019–2020 Rwanda Demographic and Health Survey (DHS) reported a 30% discontinuation rate among women within the first year of use. This study analyses predictors of discontinuation using this DHS data, with the goal of strengthening Rwanda’s family planning programs.

**Methods:**

Data from the 2019-20 Rwanda DHS (14,634 women aged 15–49) was examined. A two-stage sampling design informed the survey. Life table methods and Cox proportional hazard models were used to analyze discontinuation rates, median usage duration across contraceptive methods, and the influence of demographic and other factors.

**Results:**

Results indicated a progressive rise in contraceptive discontinuation over different period: 16.69% at 6 months, 29.29% at 12 months, and 47.21% at 24 months. Pills and male condoms showed higher discontinuation probabilities early on. While injectables and LAM initially showed lower discontinuation, rates rose significantly by the 24th month. Health concerns and side effects were the primary reasons cited for discontinuation. The Cox proportional hazards analysis revealed significant factors influencing discontinuation: contraceptive method, desire for pregnancy, husband’s disapproval, access/availability, and the desire for a more effective method.

**Conclusion:**

This study highlights substantial contraceptive discontinuation rates in Rwanda, particularly for pills and injectables. Method type, health concerns, side effects, and method failure were associated with discontinuation. Interventions should focus on improving contraceptive continuation and investigating alternative methods with lower discontinuation tendencies.

## Background

Contraceptive discontinuation remains a significant barrier to effective family planning (FP) in Rwanda. Despite substantial progress in the adoption of modern contraceptives for nearly a decade until the late 2000s [1], stagnation in uptake has become evident. The 2019–2020 Rwanda Demographic and Health Survey (DHS) found a concerning 30% discontinuation rate within 12 months of use, mirroring an increase from the 28% reported in the 2014/15 DHS. Of particular concern are the high discontinuation rates of contraceptive pills and injections, the most widely used methods among married women. Understanding the complexities of discontinuation is essential for adapting FP programs to meet these needs.

Discontinuation encompasses the termination of contraceptive use episode while at risk of unintended pregnancy. It manifests in several ways, including abandonment, method switching, and method failure [2–4]. While approximately one-third of discontinuations can be attributed to childcare responsibilities [5, 6], a substantial portion stems from contraceptive failure or method-related issues [7–9]. Unintended pregnancies resulting from discontinuation pose severe health risks for women in developing countries, often due to unsafe abortions and high maternal mortality rates.

Research has established several influential demographic and social factors associated with contraceptive discontinuation across various contexts. Age plays a significant role [10, 11], with younger women demonstrating higher discontinuation rates while still requiring protection [12–14]. Factors like inexperience, misinformation, and negative experiences with FP providers contribute to ineffective contraceptive use among adolescents [11, 15]. Young girls may opt for less reliable methods due to fears of sterility [16], or even discontinue or share contraceptive pills due to negative interactions with healthcare providers [15]. Age also influences fertility desires, with the wish for additional children and current number of children strongly predicting discontinuation [12].

A woman’s education level is another influencing factor. Comparative analyses of DHS data from multiple countries demonstrate an inverse relationship between discontinuation and years of education [7]. Similar findings, though not definitively explanatory, are reported by Hubacher et al. [8]. Limited contraceptive knowledge and inadequate access to family planning services led to suboptimal contraceptive use patterns [17, 18].

Evidence reveals a complex relationship between discontinuation rates, method switching, and women’s education levels. While well-educated women may be more likely to switch methods [11], some studies indicate a weaker correlation between discontinuation and education [13]. This relationship likely depends on the availability of contraceptive choices and the underlying reasons for discontinuation. Greater access to various methods empowers women to switch to more effective options [10]. Additionally, women with higher education often possess better information and are less influenced by misconceptions, leading them to prefer more effective methods [11].

Beyond education, socioeconomic factors like employment status, location (e.g., urban/rural), and communication within relationships also influence contraceptive discontinuation [10, 14]. Employed women tend to demonstrate lower discontinuation rates, likely due to their desire to maintain employment and potential for greater access to FP information and decision-making power. Furthermore, the type of employment matters. A Nepalese study found that women working in agriculture had higher discontinuation rates for pills and injectables [9].

Disparity in access presents logistical hurdles for women seeking consistent contraceptive use in resource-limited settings. Long distances to work, frequent visits for injections or pills, and the distance to health facilities create significant inconvenience. A Nepalese study established an inverse relationship between discontinuation and distance to health services [9]. Additionally, place of residence impacts discontinuation rates. Rural/urban divides in information access, service availability, and staffing within FP programs can create barriers for women in rural areas [1].

Rwanda’s challenges with contraceptive discontinuation mirror those found in other low- and middle-income countries. Studies in Ethiopia [10] and Nepal [9] report discontinuation rates at 28.9% within six months and 35.6% for pills / 46.6% for injectables respectively. Younger women demonstrate greater discontinuation tendencies across contexts like Bangladesh [11] and India [14]. The correlation between discontinuation and education level is evident internationally [7] and specifically in Pakistan [18]. Finally, the higher likelihood of rural women to discontinue their contraceptive use is a trend observed both in Rwanda [1] and other low/middle-income countries [17].

Despite progress, Rwanda’s modern contraceptive uptake has stagnated. While the Ministry of Health targeted 62% uptake among married women by 2015, a concerning 30% discontinuation rate within 12 months remains an obstacle [1]. Policymakers require in-depth information to tailor and improve FP programs effectively. This study aims to illuminate the predictors of contraceptive discontinuation in Rwanda using the 2019/20 Rwanda Demographic and Health Survey (DHS) data.

## Methods

### Data source

The 2019-20 Rwanda Demographic and Health Survey (DHS) employed a two-stage sampling design to collect comprehensive reproductive health data from 14,634 women aged 15–49. Clusters were selected from enumeration areas in the first stage, followed by household selection within these clusters. Women residing in or visiting sampled households on the survey night were interviewed.

The DHS questionnaire features a reproductive calendar section with a two-column format, documenting events over five years prior to the interview. The first column records reproductive events (contraceptive use/non-use, pregnancies, births, terminations). The second column notes reasons for any contraceptive discontinuation. Specific codes denote each event. Continuous use of the same method is inferred from consecutive positions with identical codes, forming an episode of use. Discontinuation is indicated by code changes in subsequent positions.

Importantly, the DHS program provides a codebook that precisely defines the reproductive calendar codes. The 2019-20 Rwanda DHS codebook was essential for analyzing this data and calculating contraceptive discontinuation rates. Our analysis strictly follows these coding standards for accurate representation and interpretation of the survey’s reproductive behaviour data.

### Variables

This study focuses on discontinuation rates and the median duration of contraceptive method use as outcome variables to describe patterns and trends in discontinuation. Our analysis employed a comprehensive range of demographic, socioeconomic, and contextual variables selected for their theoretical and empirically established relevance.

Specifically, these included, demographic factors: Age, education level, and number of living children. Socioeconomic factors: Occupation and access to FP services from health facilities and contextual factors: Place of residence (urban/rural) and interpersonal dynamics, including the partner’s desired number of children and experience of violence. This multifaceted approach aims to capture the complex and interrelated factors influencing contraceptive use and discontinuation.

### Statistical methods

This study employed a two-part statistical approach to analyze contraceptive discontinuation patterns and their predictors.

1. **Life Table Methods**: Discontinuation rates and trends over time (6, 12, and 24 months) were estimated using life table methods. This approach is well-suited for addressing right censoring, common when analyzing ongoing contraceptive use at the time of a survey [19, 20]. Life tables consider all use episodes with discontinuation as the event of interest.
2. **Cox Proportional Hazards Model**: To identify the most influential predictors of discontinuation, we used a stepwise selection process within a Cox proportional hazards model to estimate hazard ratios for each period. Starting with a comprehensive model “Full model” including all potential predictors, variables with low explanatory power (*p*-value > 10%) were iteratively removed. This resulted in a “reduced model” retaining the most statistically significant factors (*p*-value < 5%, 95% CI). This approach provides a focused and informative representation of the key determinants of contraceptive discontinuation [21–23].

## Results

This section examines contraceptive discontinuation patterns over 6, 12, and 24 months. Life tables and a survival analysis model with hazard ratios were used to assess discontinuation risk. Table 1 summarizes discontinuation rates by method, reason, and timeframe, along with the number of use episodes for each method. The discontinuation rate nearly doubled between 6 and 12 months, reaching 47.2% by 24 months, highlighting a significant trend across all methods and reasons.

The contraceptive pill exhibited the highest discontinuation rates throughout the analysis (35.6% at 6 months, 50.8% at 12 months, 68.6% at 24 months) and the most frequent method switching. Despite accounting for two-thirds of use episodes, injectables showed a doubling of discontinuation between 6 and 12 months, reaching 57.7% by 24 months. Implants consistently displayed the lowest discontinuation rate across all periods. Male condoms consistently showed a high discontinuation rate. Table 1 (2019/20 Rwanda DHS) provides detailed percentages of contraceptive methods and reasons for discontinuation.

Life table analysis, which excluded continued users from censoring and recalling, revealed significant patterns in discontinuation rates over time: Discontinuation increased notably, reaching 16.69% at 6 months, 29.29% at 12 months, and 47.21% at 24 months. Users of pills (35.66%) and male condoms (24.62%) demonstrated the highest likelihood of discontinuation within 6 months. In 12 months, the discontinuation rates rose for pills (50.89%), male condoms (43.58%), injectables (36.9%), and LAM (34.06%). While, in 24 months, the pills (68.66%), LAM (59.65%), male condoms (58.88%), and injectables (57.75%) continued to show the highest discontinuation probabilities.

**Table 1:** Life table: 6-month, 12-month, and 24-month periods discontinuation rates by contraceptive methods and reasons for discontinuation

Methods	Method failure	Desire to become pregnant	Other fertility-related	Side Effects	Other method related	Other/ DK	In Need	Switching	Episodes
6-Month Period									
Pill	2.45	2.56	1.75	11.39	15.97	1.54	35.66	14.82	1392
Injectables	0.88	2.60	2.42	9.46	3.58	1.26	20.19	4.12	2839
Implant	0.31	0.27	0.20	1.71	0.09	0.24	2.82	0.41	2742
Male condom	1.02	3.55	3.06	0.69	11.76	4.53	24.62	8.90	588
LAM	3.10	6.67	0.00	0.00	5.28	0.43	15.48	3.27	239
Other	2.44	2.39	1.44	1.63	2.76	1.75	12.40	1.63	614
All methods	1.14	2.00	1.49	5.78	5.06	1.22	16.69	4.82	8413
12-Month Period									
Pill	4.83	5.37	2.43	16.18	19.65	2.43	50.89	19.34	1392
Injectables	1.46	5.73	3.71	17.07	6.61	2.32	36.90	7.94	2839
Implant	0.44	0.83	0.41	4.64	0.14	0.35	6.81	1.23	2742
Male condom	2.77	8.82	3.77	0.72	19.83	7.66	43.58	14.33	588
LAM	10.96	12.02	0.71	0.90	8.18	1.29	34.06	4.73	239
Other	7.34	5.56	1.57	3.04	4.55	1.81	23.86	1.94	614
All methods	2.52	4.55	2.22	10.39	7.58	2.04	29.29	7.66	8413
24-month Period									
Pill	8.97	11.28	4.06	20.25	21.68	2.43	68.66	21.17	1392
Injectables	3.39	11.62	5.01	24.76	10.14	2.84	57.75	11.07	2839
Implant	1.23	2.42	0.81	12.05	0.61	0.65	17.77	3.14	2742
Male condom	4.89	14.64	5.13	1.36	24.38	8.47	58.88	17.11	588
LAM	21.21	22.64	0.71	0.90	10.33	3.85	59.65	5.54	239
Other	12.68	12.21	1.83	5.38	6.22	2.57	40.90	3.25	614
All methods	5.17	9.76	3.29	16.43	10.05	2.51	47.21	10.11	8413

Other/DK includes all responses other than those listed above, and women who said they did not know or remember why they discontinued

Switching increased over time for all methods: 4.82% (6 months), 7.66% (12 months), and 10.11% (24 months). Pills and male condoms consistently showed the highest switching rates throughout the study periods. Health concerns and side effects were the most common reasons for discontinuation, with rates of 5.78% (6 months), 10.39% (12 months), and 16.43% (24 months).

Dominant reason, accounting for 24.88% (6 months), 42.55% (12 months), and 64.7% (24 months) of discontinuations. Increased notably over time, with rates of 10.2% (6 months), 27.8% (12 months), and 52.37% (24 months). Also rose across the study period: 33.15% (6 months), 49.51% (12 months), and 61.28% (24 months). Table 1 provided a descriptive overview of discontinuation patterns by method and timeframe. To identify specific factors driving discontinuation, Table 2 presents a more in-depth statistical analysis.

Our analysis revealed key factors influencing contraceptive discontinuation: A positive association exists between the number of contraceptive use episodes and the hazard of discontinuation (HR = 1.05, 95% CI: 0.077, 0.113). This indicates that women with multiple contraceptive events (e.g., repeated pill cycles or injections) were more likely to discontinue. The results showed a decreased risk of discontinuation for users of the following methods: Pills (HR = 0.389, 95% CI: 0.217, 0.560); Injectables (HR = 0.291, 95% CI: 0.167, 0.414); Implant (HR = 0.365, 95% CI: 0.194, 0.535); Male Condom (HR = 0.405, 95% CI: 0.172, 0.638) and Other methods (HR = 0.601, 95% CI: 0.241, 0.961).

Our Cox proportional hazards model revealed several factors significantly influencing the risk of contraceptive discontinuation: Desire for pregnancy had the strongest impact (HR = 1.607, 95% CI: 1.364, 1.850), signifying a substantial increase in discontinuation likelihood. Husband disapproval (HR = 2.43, 95% CI: 2.180, 2.679) highlighted the critical role of partner support in contraceptive decision-making. Access and availability limitations (HR = 2.049, 95% CI: 1.699, 2.4) underscored the challenges hindering consistent contraceptive use.

**Table 2:** Hazard ratio for survival analysis model of the contraceptive discontinuation

	Full					Reduced				
Model	Estimate	HR	P-val.	(95% CI)		Estimate	HR	P-val.	(95% CI)	
**Woman’s age**										
15–24 years	Ref									
25–34 years	1.142	0.133	0.247	-0.092	0.358					
35–49 years	0.907	-0.097	0.487	-0.372	0.177					
Event Number	1.057	0.056	0.000	0.034	0.079	1.099	0.095	0.000	0.077	0.113
**Contraceptive Method**										
No Using	Ref									
Pill	1.426	0.355	0.002	0.134	0.575	1.475	0.389	0.000	0.217	0.560
Injectables	1.114	0.108	0.186	-0.052	0.267	1.337	0.291	0.000	0.167	0.414
Implant	1.381	0.323	0.006	0.094	0.552	1.440	0.365	0.000	0.194	0.535
Male condom	1.395	0.333	0.029	0.034	0.632	1.499	0.405	0.001	0.172	0.638
LAM	1.585	0.461	0.311	-0.431	1.352	1.977	0.682	0.097	-0.123	1.486
Other	2.062	0.724	0.001	0.292	1.156	1.824	0.601	0.001	0.241	0.961
**Residence**										
Urban	Ref									
Rural	0.841	-0.173	0.077	-0.365	0.019					
**Woman’s education**										
No education	Ref									
Primary	0.764	-0.269	0.184	-0.667	0.128					
Secondary	0.775	-0.255	0.162	-0.611	0.102					
Higher	0.709	-0.344	0.086	-0.736	0.048					
**Province**										
Kigali City	Ref									
Eastern	1.076	0.073	0.572	-0.181	0.328					
Northern	1.043	0.042	0.763	-0.234	0.319					
Southern	0.973	-0.027	0.836	-0.284	0.230					
Western	0.963	-0.038	0.782	-0.305	0.229					
**Reason for Discontinuation**										
Become Pregnant	Ref									
Wanted to have a child	5.168	1.643	0.000	1.319	1.966	4.988	1.607	0.000	1.364	1.850
Husband Disapproved	13.948	2.635	0.000	2.305	2.966	11.35	2.430	0.000	2.180	2.679
Side Effects	0.244	-1.413	0.000	-2.053	-0.772	0.345	-1.063	0.000	-1.476	-0.651
Health Concerns	0.349	-1.054	0.145	-2.472	0.364	0.230	-1.471	0.012	-2.625	-0.317
Access/availability	10.585	2.359	0.000	1.901	2.818	7.761	2.049	0.000	1.699	2.400
Wanted more effective	1.655	0.504	0.073	-0.047	1.055	2.292	0.829	0.000	0.461	1.197
Inconvenient to use	0.385	-0.953	0.000	-1.268	-0.639	0.325	-1.123	0.000	-1.359	-0.887
**Parity**										
<3 Children	Ref									
4–6 Children	1.158	0.147	0.128	-0.042	0.335					
7 + Children	1.267	0.237	0.112	-0.055	0.529					
**Wanted the last child**										
Wanted then	Ref									
Wanted later	1.023	0.023	0.764	-0.128	0.174					
No more	0.958	-0.043	0.673	-0.242	0.156					
**Visited by Family Planning Worker**										
Not Visited	Ref									
Visited	0.947	-0.054	0.467	-0.201	0.092					
**Family Planning at Health Facility**										
Not Informed	Ref									
Informed	1.021	0.092	0.175	-0.041	0.224					
**Women’s employment**										
Unemployed	Ref									
Employed	1.053	0.021	0.889	-0.273	0.315					
Self-employed	0.678	0.052	0.736	-0.248	0.351					
**Household Wealth**										
Lowest third quintile	Ref									
Highest two quintile	0.935	-0.067	0.383	-0.218	0.084					
**Marital status**										
Unmarried	Ref									
Married	1.096	-0.389	0.000	-0.606	-0.171	0.370	-0.994	0.000	-1.149	-0.839

The desire for a more effective method showed a trend towards increased discontinuation, but with a less significant impact (HR = 0.829, 95% CI: 0.461, 1.197). Surprisingly, both side effects (HR = -1.063, 95% CI: -1.476, -0.651) and health concerns (HR= -1.471, 95% CI: -2.625, -0.317) were negatively associated with discontinuation. This suggests that while these factors are common, they might lead to method switching rather than complete discontinuation.

Inconvenience with a method was negatively associated with discontinuation (HR = -1.123, 95% CI: -1.359, -0.887). This suggests users may adapt to initial inconvenience over time or find ways to overcome these challenges. Married women showed a slightly reduced risk of discontinuation (HR = -0.994, 95% CI: -1.149, -0.839). This could imply greater stability in contraceptive decision-making or access to resources within marriage.

## Discussion

This study highlights the significant challenge of contraceptive discontinuation in Rwanda, with rates doubling between 6 and 12 months and reaching 47.2% by 24 months. Our findings identify the pill as the method with the highest discontinuation rates across all timeframes, followed by injectables and implants. Implants consistently showed the lowest discontinuation rates, while male condoms also demonstrated high discontinuation.

The life table analysis corroborates these trends, emphasizing the substantial issue of discontinuation. Pill and male condom users displayed the greatest likelihood of discontinuation within the first 6 months. The observed switching rate exceeding 10% at 24 months, primarily among pill and male condom users, further highlights the challenges in sustaining contraceptive use. These findings align with previous studies like Ali et al. [24], underscoring the broader context of discontinuation in Sub-Saharan Africa.

The Cox proportional hazards analysis reveals health concerns and side effects as the most common reasons for discontinuation, with their impact increasing over time. The pill, injectables, and male condoms were particularly associated with a higher risk of discontinuation. Additionally, the desire for pregnancy, husband disapproval, and issues with access and availability significantly contribute to discontinuation. Interestingly, factors like age, education, and wealth did not demonstrate a strong association in our analysis.

These findings underscore the need for comprehensive strategies to address contraceptive discontinuation. Targeted education addressing concerns about side effects, such as menstrual irregularities, could improve method acceptance and continuation, as suggested by prior research [25]. Additionally, it’s important to support current users in minimizing method failure and facilitate informed switching to alternative methods if discontinuation occurs [26].

## Conclusions

This study highlights the significant challenge of contraceptive discontinuation in Rwanda, particularly for pills and injectables. Rates nearly doubled within the first year, reaching 47.2% by 24 months. While the implant demonstrated the lowest discontinuation rate, health concerns, side effects, method failure, and switching emerged as key reasons for discontinuation across methods.

Targeted interventions addressing the specific challenges of pills and injectables are essential. This includes enhanced education and support to manage side effects and reduce method failure. Additionally, exploring alternative methods like the implant for suitable users could prove beneficial. Comprehensive strategies addressing method-specific issues will promote informed decision-making and sustained contraceptive use. These actions are crucial for policymakers, healthcare providers, and public health professionals in Rwanda. By reducing discontinuation, women will be empowered to make informed reproductive health choices, potentially reducing unintended pregnancies.

## Data Availability

The manuscript has fully used DHS Datasets and free available upon request from their website https://dhsprogram.com/

## Abbreviations

CI: Confidence Interval
DHS: Demographic and Health Survey
FP: Family Planning
HR: Hazard Ratio
LAM: Lactational Amenorrhea Method
